# Intra-arterial catheter incidentally placed in the median artery in a patient with an anatomical variation of the radial artery

**DOI:** 10.1186/s40981-022-00588-3

**Published:** 2022-12-12

**Authors:** Mitsuhiro Matsuo, Satoru Honma

**Affiliations:** 1 Department of Anesthesiology, University of Toyama, 2630 Sugitani, Toyama, 930-0194 Japan; 2 Anatomy II, Kanazawa Medical University, Uchinada, Japan

**Keywords:** Anatomy, Arterial catheter, Medical safety

To the Editor

The median artery is a persistent primitive artery that accompanies the median nerve and supplies the nerve and the hand [1]. We herein describe a case in which an arterial catheter was incidentally inserted into the median artery in a patient with a superficial radial artery. Fortunately, the patient did not develop a neuropathy; however, puncture of the median artery is associated with a high risk of median nerve injury. Therefore, anesthesiologists must be familiar with the anatomical variations of the median and superficial radial arteries in the forearm and wrist to achieve safe cannulation.

A 61-year-old man was scheduled for open hepatectomy for hilar cholangiocarcinoma. After induction of anesthesia, an anesthesiology fellow was unable to palpably identify the artery on the radial side of the right distal forearm (circle in Fig. 1). She located an artery on the distal forearm using color Doppler and inserted a 20-gauge catheter on the first attempt. The blood was able to be withdrawn via that catheter, and the arterial waveform was normal. An attending anesthesiologist found that, however, the arterial catheter was unexpectedly placed in the median artery, which runs between the flexor carpi radialis tendon and the palmaris longus tendon (Fig. 1). The color Doppler ultrasonography confirmed that the artery ran anterior to the median nerve. After surgery, the patient denied any palmar paresthesia and any difficulty of finger–thumb opposition.

**Fig. 1:**
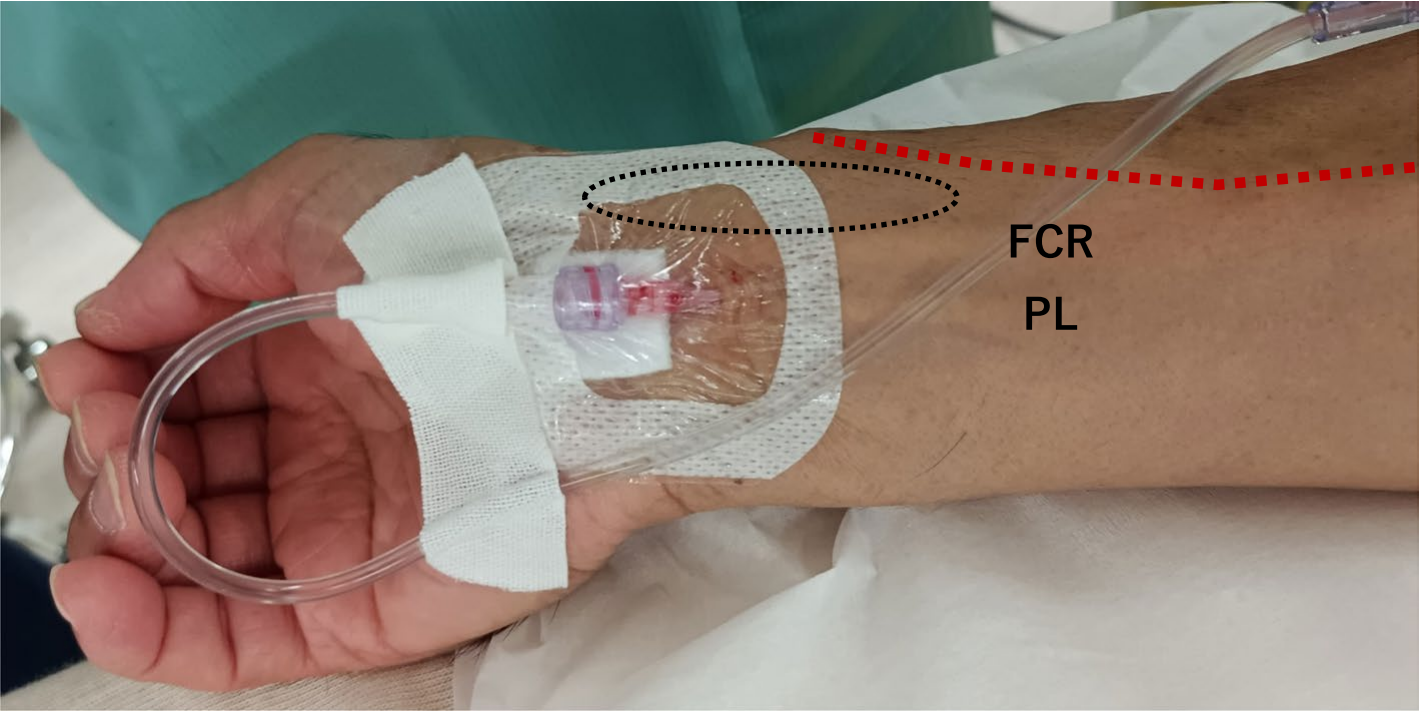
Incidental arterial cannulation of the median artery. An arterial catheter was placed between the flexor carpi radialis (FCR) tendon and the palmaris longus (PL) tendon, at the concordance of the running course of the median nerve. The superficial radial artery, an anatomical variation of the radial artery, was dominant to the radial artery in our patient (red line). Therefore, the pulse was difficult to palpate in the distal forearm where the radial artery usually runs (circle)

The median artery is an embryonic structure that typically regresses by the eighth week of gestation [1]. Moreover, the prevalence of the median artery declines with age, even after birth [2]. An ultrasonographic study showed a persistent median artery in 26% of adults, and the mean diameter of the artery was 1.1 mm (range, 0.5–1.7 mm) [3]. Although incidental cannulation of the median artery was reported in a 1-year-old child [4], this is the first report in an adult. The median artery runs along the anterior aspect of the median nerve [5]. Therefore, penetration of the posterior wall of the median artery is associated with a risk of nerve injury.

The superficial radial artery, an anatomical variant of the radial artery that runs over the anatomical snuffbox, has been reported at a frequency of 0.5 to 1.0% [6]. Our patient had a superficial radial artery with a normal origin of the radial artery, which runs subcutaneously and crosses over the tendons, defining the snuffbox at the wrist level (red line in Fig. 1). The existence of a superficial radial artery implies difficulty detecting the normal radial pulse at the wrist level [7]. Thus, the presence of a radial artery variant was thought to be the main reason for the incidental cannulation in this case.

In conclusion, this case provides two learning points. The first is that the presence of a superficial radial artery results in difficulty detecting the normal radial pulse at the wrist, and the second is that puncturing the median artery is associated with a high risk of injury to the median nerve.

## Data Availability

Data sharing is not applicable to this article, as no datasets were generated or analyzed for the report.
